# Constructing Others' Beliefs from One's Own Using Medial Frontal Cortex

**DOI:** 10.1523/JNEUROSCI.0011-21.2021

**Published:** 2021-11-17

**Authors:** Nils Kolling, Marius Braunsdorf, Suhas Vijayakumar, Harold Bekkering, Ivan Toni, Rogier B. Mars

**Affiliations:** ^1^Wellcome Centre for Integrative Neuroimaging, Department of Psychiatry, University of Oxford, Oxford OX3 7JX, United Kingdom; ^2^Donders Institute for Brain, Cognition and Behaviour, Radboud University Nijmegen, 6525 AJ Nijmegen, The Netherlands; ^3^Wellcome Centre for Integrative Neuroimaging, Centre for Functional MRI of the Brain, Nuffield Department of Clinical Neurosciences, John Radcliffe Hospital, University of Oxford, Oxford OX3 9DU, United Kingdom

**Keywords:** belief, cognitive neuroscience, evidence, medial frontal cortex, social interaction, social neuroscience

## Abstract

Many daily choices are based on one's own knowledge. However, when predicting other people's behavior, we need to consider the differences between our knowledge and other people's presumed knowledge. Social agents need a mechanism to use privileged information for their own behavior but exclude it from predictions of others. Using fMRI, we investigated the neural implementation of such social and personal predictions in healthy human volunteers of both sexes by manipulating privileged and shared information. The medial frontal cortex appeared to have an important role in flexibly making decisions using privileged information for oneself or predicting others' behavior. Specifically, we show that ventromedial PFC tracked the state of the world independent of the type of decision (personal, social), whereas dorsomedial regions adjusted their frame of reference to the use of privileged or shared information. Sampling privileged evidence not available to another person also relied on specific interactions between temporoparietal junction area and frontal pole.

**SIGNIFICANCE STATEMENT** What we know about the minds of others and how we use that information is crucial to understanding social interaction. Mentalizing, or reading the minds of others, is argued to be particularly well developed in the human and crucially affected in some disorders. However, the intractable nature of human interactions makes it very difficult to study these processes. Here, we present a way to objectively quantify the information people have about others and to investigate how their brain deals with this information. This shows that people use similar areas in the brain related to nonsocial decision-making when making decisions in social situations and modify this information processing by the knowledge about others use these to modify their information processing according to the knowledge of others.

## Introduction

When making social predictions, a good first step is to ask “what would I do?” However, without considering how others differ in their beliefs and knowledge, such egocentric predictions can be problematic, particularly when not all knowledge is shared. Such mentalizing challenges are often discussed in the context of autism, and are a feature of social interactions in daily life. To make better predictions about others, it is important to know what aspects of one's own world view is privileged (i.e., unknown by another person) and then ignore that information. This is why we wanted to understand how the brain (1) marks some information as privileged and other as shared and (2) selectively uses only the relevant information when making predictions about the world or other people. By understanding the neural and cognitive mechanisms of constructing one's own and others' beliefs to make different predictions, we can also learn more general truths about how the brain allows us to entertain multiple beliefs interchangeably for social and personal purposes alike.

We addressed these issues by using a sequential evidence accumulation paradigm. The neural implementation and the algorithm supporting this type of decision-making have been extensively characterized, both in macaque single-neuron activity (Yang and Shadlen, 2007) and in human neural activation (Gould et al., 2012). Generally, areas of the parietal-frontal attentional system scale their activity with evidence accumulated in favor of a possible alternative. Frontal regions, including regions on the medial wall, are generally involved in the decision process (i.e., transformation of this information into a response) (Thoenissen et al., 2002) similarly to value-based decision paradigms (Boorman et al., 2009). Here, we use a social variant where participants had to sequentially accumulate evidence about the state of the world as they knew it and about a partner's belief of the world, followed by decisions based on either of these two sources of information. This “social evidence accumulation” paradigm allowed us to disentangle what participants knew to be true from what they knew the partner knew.

Some have suggested that parts of cingulate cortex very close to the above-mentioned decision-related regions, including the gyral part of the mid-cingulate cortex, have a unique role in processing social information (e.g., Rudebeck et al., 2006; Apps et al., 2013), whereas others instead emphasize the similarity between social information processing and personal decision-making in dorsal anterior parts of medial frontal cortex (MFC) (Isoda and Noritake, 2013). As a case in point, a task in which participants were following instructions from a director with a known but different perspective to the subject evoked activation in the superior part of the anterior MFC (Dumontheil et al., 2010), but these activations were not explicitly contrasted with self-related decision-making. Activation of more posterior dorsomedial regions has been associated with inhibition, including inhibition of representations of how the subject knows the world to be (Gagnepain et al., 2014). Because we asked our participants to make both social and personal predictions, we can explicitly compare activity in both types of judgment.

In short, this study leverages the well-known computational paradigm of evidence accumulation to understand a core element of human social cognition. Specifically, we are interested in how people track information that is relevant for their own judgment of the world while concurrently considering what another person knows about that information. Furthermore, we wanted to understand how two people construct predictions and make choices from the information they received and from what they know about another person's information state.

## Materials and Methods

### Participants

A total of 28 participants (age range 19-33 years, mean 24.95 years; 14 female) were included in this study. As this is a novel paradigm in social decision-making, the study could be considered exploratory and it was not possible to perform a power calculation based on previous effect sizes in this context. All participants were righthanded, reported no history of neurologic or psychiatric disorders, and had normal or corrected-to-normal vision. Participants provided written informed consent before the start of the experiment; they received a compensation of €10 per hour for participation, which lasted 2.5 h for training and scanning. All procedures were approved by the relevant ethics authority (CMO Arnhem-Nijmegen) in accordance with the Declaration of Helsinki.

### Task

During the task, participants viewed a screen where they witnessed the scene from a first-person point of view. They were facing a virtual other person seated behind a table opposite the participant. In between the participant and the other person, a screen was placed on the table. In the first phase of the trial (sampling phase), parts of the screen consisting of a square one-ninth of the screen in size sequentially opened up, revealing a cloud of red and blue dots. Per trial, 3 (12.5% or trials), 4 (75%), or 5 (12.5%) parts of the screen opened up. Each open section remained visual for 1.5-2.5 s (normal distribution with mean = 2.0 s). The open sections had either a white or light gray background, indicating whether the cloud of dots was available to both the participant and the other (shared sample) or only to the participant and not to the other (occluded sample).

Occluded and shared samples were drawn from independent distributions but we made sure that occluded samples were more likely to have strong evidence in either direction. This ensured more trials where the occluded evidence had a significant influence on the participant's response than would be expected if the same sampling distributions were used for shared and occluded samples, since shared samples outnumber occluded ones. This also led to participants making different choices for themselves and the other person in 36.7% of all trials. There was no bias as to how many dots of each color were displayed on each screen; the number of dots on a single screen was never >45 but otherwise covered the whole spectrum of blue/red ratios.

After viewing all samples, in the next phase of the trial (decision phase), participants were asked to indicate whether they had seen more red or blue dots in total (Self Decision) and whether the confederate had seen more red or blue dots (Other Decision) by pressing a button corresponding to the position of the word “red” or “blue” on the screen. The mapping between answers and buttons was determined randomly on each trial to avoid motor preparation before the decision screen. After the button press was recorded, the corresponding word on the screen was framed in the respective color.

In the final part of the trial (feedback phase), participants received feedback on the choice the other person actually made in that trial by highlighting the word indicating that color on the screen for 500 ms. The behavior of the other person was determined using a softmax function of evidence against probability of choosing the correct answer. Softmax inverse temperature varied across “good” other persons (inverse temperature = 0.3) and “bad” other persons (0.03). Thus, the other person's choice was not 100% predictable encouraging participants to observe and learn about the other's ability level. This inferred ability was probed every 10 trials (judgment phase), when participants were presented with a 10 point scale ranging from “She/He is doing very badly” to “She/He is doing very well” in which the participants used the buttons to indicate how well they believed the participants were doing. The other person's identity was changed every 40 trials; all participants encountered two good and two bad others. The appearance and order of the avatars were randomized across participants.

### Training

Directly before the scanning session, participants completed an extensive four-part training on a personal computer in a dedicated behavioral testing laboratory to introduce them to the different aspects of the task. During the first part (10 trials), participants performed the task with only nonoccluded screens and were required to indicate whether they had seen more red or more blue dots (i.e., the Self Decision); in contrast to the later experiment, they received feedback on the accuracy of their choice. The second part (10 trials) closely resembled the first, but feedback was absent, as it would be during the scanning session. In the third training session (50 trials), occluded screens were introduced and participants were also asked to indicate the choice of the other (i.e., the Other Decision), who was seen but inactive during the first two parts. During this part, the behavior of the other was perfect so the feedback participants received on the behavior of their confederate served as feedback on the other decision. The last part (50 trials) had all the aspects of the actual experiment in it, with two different others possessing two different ability levels. In contrast to the later experiment, participants were asked to rate the ability of those others every 5 instead of every 10 trials. The ability levels also differed from those in the final experiment to avoid carryover learning effects.

### Experimental session

**Figure 1.**
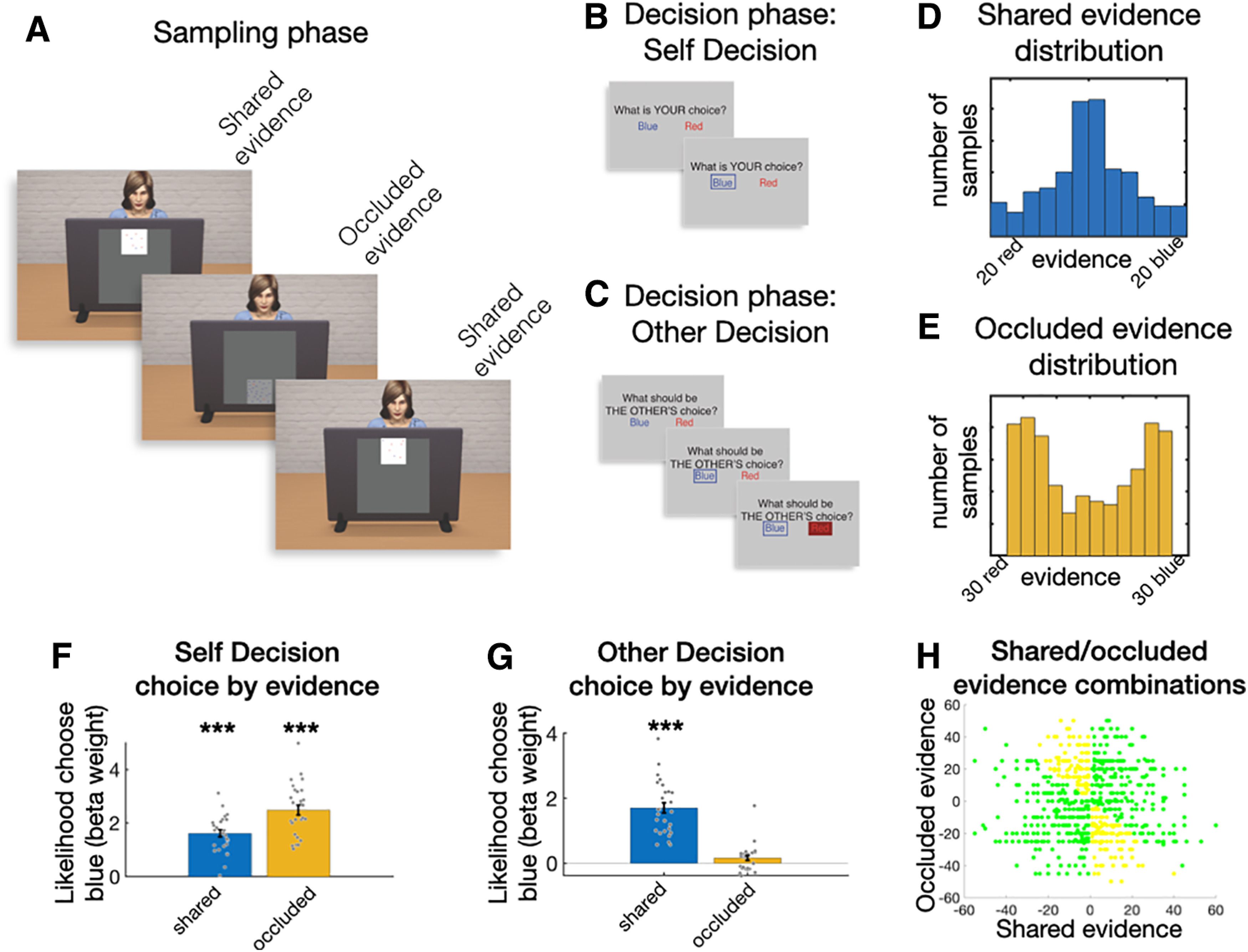
Experimental design and behavioral results. Each trial consisted of a sampling phase and a decision phase. ***A***, During the sampling phase, participants were sequentially presented with open windows that showed part of a blue and red dot cloud. Some windows were open to both the participant and a confederate avatar projected opposite of the participant on the screen (shared evidence, white). Some were only visible to the participants and thus were privileged information (occluded evidence, gray). ***B***, The decision phase started with a Self Decision in which participants had to indicate whether they thought more blue or more red dots were present in the trials. ***C***, Then followed an Other Decision in which the participants had to rate what they believed the confederate would respond based on the shared evidence. This was followed by feedback on what the confederate actually chose, and allowed the participant to form an opinion of the ability of the confederate. Distributions of shared (***D***) and occluded (***E***) evidence differed, to ensure that occluded evidence had a notable effect on behavior. ***H***, All observed shared/occluded evidence combinations. Yellow points represent combinations that should lead to a difference between self and other choices. For the behavioral data, binomial regressions show the influence of evidence presented in shared and occluded screens on choice behavior in the (***F***) Self Decision and (***G***) Other Decision.

The experimental session directly followed the training. Participants conducted the experiment in a supine position, while we collected fMRI data (for specifics, see next section). The task consisted of 160 trials. Participants played with four different others presented in blocks, amounting in 40 trials per other. Those others possessed two different ability levels. Other identity and ability level assignment were randomized to avoid confounds associated with their appearance. Each trial, participants indicated whether they perceived more red or blue (see Fig. 1*B*, Self Decision); subsequently, they indicated what their other should say based on the information available to them (see Fig. 1*C*, Other Decision).

To decorrelate fMRI regressors between self and other decisions, the fixation cross between the two questions ranged between 2.5 and 6.5 s (mean = 3.5) following a Poisson distribution. We provided a possibility for learning about the other's ability by presenting feedback on its actual behavior for participants after they decided on the other's answer. Every 10 trials (4 times per confederate), participants indicated their opinion about the ability of the other on a 1-10 scale.

Participants watched a projection of the experiment screen through a mirror placed on top of the MRI receive coil. For each participant, we adjusted the viewing distance individually, according to appropriate head positioning in the scanner. The complete scanning session took ∼90 min: 1 h experimental session, leaving us 30 min for preparation and high-resolution structural scan (see Data acquisition).

### Data acquisition

MRI data were acquired on a 3T Siemens PrismaFit, using a 32 channel head coil. Functional scans were collected in a single run using a gradient EPI sequence, multiband 4, with TR = 1.5 s, TE = 32 ms, flip angle = 75 degrees, and voxel resolution of 2 × 2 × 2 mm. Structural scans were acquired using a T1-weighted MP-RAGE sequence, 1 × 1 × 1.3 mm voxels. All scans provided whole-brain coverage, with EPI slices angled downward in the posterior direction.

### Behavioral data analysis

To analyze the choices participants made, we used logistic multiple regressions implemented in MATLAB (glmfit). We ran three separate regression analyses. (1) We analyzed the Self Decisions predicting blue choices and including as regressors the relative evidence for blue dots in the occluded and shared samples separately. As there was more than one sample for each in most trials, we summed all evidence within the samples of one category as follows:

$$relativeblue\_sharedEvidence=\overset{x=n}{\underset{x=1}{\sum }}relativeblue\_sharedsample_{x}$$

where *n* is the shared sample number

$$relativeblue\_occludedEvidence=\overset{x=n}{\underset{x=1}{\sum }}relativeblue\_occludedsample_{x}$$

where *n* is the occluded sample number

Thus, the GLM for the Self Decision was as follows:

$$y_{selfchoices}=\beta_{0}+\beta_{1}\left(relativeblue_{sharedEvidence}\right)+\beta_{2}\left(relativeblue_{occludedEvidence}\right)$$

We ran the same GLM for the Other Decision, except that the data were the other choices as follows:

$$y_{otherchoices}=\beta_{0}+\beta_{1}\left(relativeblue_{sharedEvidence}\right)+\beta_{2}\left(relativeblue_{occludedEvidence}\right)$$

To test whether there is a significant difference between the use of occluded evidence in the Self and Other Decision, we combined all the choices and ran a regression with decision type (self/other) as a main effect and an interaction factor with each type of evidence (shared/occluded) to test for a significant interaction term between self and other and occluded evidence as follows:

$$y_{allchoices}=\beta_{0}+\beta_{1}\left(relativeblue_{sharedEvidence}\right)+\beta_{2}\left(relativeblue_{occludedEvidence}\right)+\beta_{3}\left(DecisionType\right)+\beta_{4}\left(DecisionType\times relativeblue_{sharedEvidence}\right)+\beta_{5}\left(DecisionType\times relativeblue_{occludedEvidence}\right)$$

Furthermore, we ran a repeated-measures ANOVA to test whether the participant noticed the skills of the different avatars. We note that all task regressors were only weakly correlated (<0.3), allowing good estimation of their unique contributions.

### Imaging data analysis

DICOM images were converted to NIFTI format using MRconvert. Subsequent analyses of MRI data were performed using tools from FSL (www.fmrib.ox.ac.uk/fsl). Preprocessing of the functional data consisted of motion correction using MCFLIRT, nonbrain removal using BET, spatial smoothing using a Gaussian kernel of 5 mm FWHM, grand-mean intensity normalization of the 4D dataset by a single multiplicative factor, and high-pass temporal filtering (Gaussian-weighted least-squares straight line fitting, σ = 50.0 s). Functional images were registered to skull-stripped structural images using FLIRT; registration of structural images to MNI152 standard space was conducted using FLIRT followed by refinement using FNIRT nonlinear registration. First-level analyses were performed in each subject's native functional space, group analyses in MNI152 standard space.

The regression model for the first-level fMRI analysis in FEAT was run using FILM prewhitening and had the following regressors. First, we modeled the Main effect of each sample, Self Decision, Other Decision, and Feedback as stick functions. Then we modeled other regressors of each phase with the same duration, that is, as stick functions (implemented in FSL as duration = 0).

In the sampling phase, we had one regressor that compared occluded versus shared trials, the amount of evidence when it was an occluded sample, the amount of evidence when it was a shared sample, the overall sample number for the shared samples in the trial, and the same for the occluded samples.

In the Self Decision, we only had the Chosen relative occluded and Shared evidence (i.e., the overall shared and occluded evidence separately and signed by which color the participant had chosen in that trial). Coding evidence and value in a choice frame are common and indicate brain regions that accumulate evidence as such accumulation speed and aggregate activity are modulated by the relative evidence for the option that is ultimately chosen (as this indicates the bound an accumulator ended up integrating toward).

For the Other Decision, we had the exact same regressor model, except now coding relative evidence according to the Other Decision and not the Self Decision.

Last, we also modeled the feedback period with a binary regressor comparing correct and incorrect other predictions.

First-level results were warped to standard space for mixed-effect group analyses using Feat's FLAME 1 using automatic outlier deweighting. Thresholding was set at a conservative *Z* threshold of 2.7 and a cluster *p* threshold of 0.05. Both positive and negative effects were investigated.

Anatomical labeling was based on the FSL implementation of the connectivity-based parcellation atlases of Neubert et al. (2015) and Sallet et al. (2013) for the frontal cortex, the atlases of Mars et al. (2011, 2012) for parietal and temporoparietal cortex, the probabilistic cerebellar atlas of Diedrichsen et al. (2009), and the Juelich histologic atlas for the visual cortex (Amunts et al., 2000; Malikovic et al., 2007; Rottschy et al., 2007).

### Data and code availability

Group-level results and code implementing the analysis model will be made available online as a Data Sharing Collection on the Donders Repository and linked from the laboratory's website (www.neuroecologylab.org).

## Results

Participants performed a sequential evidence accumulation task with discrete evidence samples. Specifically, over the course of the trial's sampling phase (Fig. 1*A*), at every sampling event, the participant would be presented a specific number of blue and red dots on a two-sided virtual screen presented on the display. The participant was asked to keep track of the prevalence of red and blue dots to later be able to say whether there were more red or blue dots. Importantly, another person, represented by an avatar, was seated behind the other side of the virtual screen. Participants were told the other person was also trying to estimate blue and red dot numbers. However, while most samples were shared, some samples would only be presented to the participant and thus be occluded for the other person. This manipulation led to a discrepancy between what the participant knew about overall dot numbers and what the other person believed. After every sampling phase, participants were first asked to say whether they believed there were more red or blue dots overall (Self Decision, Fig. 1*B*). Afterwards, they had to predict what the other person (avatar) would say regarding the dots (Other Decision, Fig. 1*C*). Importantly, as the other person could not know about the occluded samples, those had to be ignored when answering the second question. The trial ended after the participant received feedback on the choice made by the other person.

While every trial provides the participants with multiple samples, throughout the manuscript, we will mostly consider them in aggregate form. We combine all shared samples of a trial into “shared evidence” and all occluded samples into “occluded evidence.” Thus, during the Self Decisions, participants should calculate the evidence based on all samples presented during a trial, combining shared and occluded evidence. In contrast, during the Other Decisions, they should calculate the evidence by taking into account only the shared evidence, ignoring the occluded evidence.

### Behavioral results

Participants' decisions were analyzed using a logistic multiple regression predicting blue choices based on shared and occluded evidence. This demonstrated that both dots presented during shared and occluded samples significantly affected choices (Fig. 1*D*; *p* values for *t* tests on both shared and occluded regression weights <0.001, Cohen's *d* > 2). In contrast, the Other Decision should ideally be driven by dots presented during the shared, but not the occluded, samples. Indeed, choices were strongly influenced by shared evidence (*p* < 0.001, Cohen's *d* = 2.08). However, those decisions were still biased by occluded evidence (*p* = 0.04, Cohen's *d* = 0.40) (Fig. 1*E*). To formally test whether the influence of the occluded evidence was nonetheless significantly lower on the Other compared with the Self Decisions, we ran a regression combining both self and other choices and included an interaction factor of decision type. This again showed significant effects of shared evidence (*p* < 0.001, Cohen's *d* = 2.43) and occluded evidence (*p* < 0.001, Cohen's *d* = 3.05). Decision type had no effect on the participants' choice (*p* = 0.08, Cohen's *d* = 0.34). However, crucially, there was a large interaction between occluded evidence and decision type (*p* < 0.001, Cohen's *d* = 1.92), suggesting, as expected, that occluded evidence was used a lot more in Self than Other Decisions.

We wanted participants to think carefully about their predictions of other people's behavior, so every 10 trials we asked them to rate how well they thought the other person was performing the task, based on the feedback they received at the end of every trial. We also made sure there was a difference in performance by blocking the experiment into four phases with a different other person each time as indicated by a changed avatar. Two avatars were “good” and two were “bad” at the task. Participants were able to detect the different levels of performance of the four avatars, rating the good ones consistently higher (mean = 7.2, SEM = 0.19) than the bad ones (5.46 ± 0.19). A repeated-measures ANOVA on the ratings with factors ABILITY (good/bad) and TIME (each of the four rating moments) indicates that participants assessed the confederates' ability very quickly and improved moderately over time, showing a main effect of ABILITY (*F*_(1,27)_ = 175.018, *p* < 0.0001, partial η^2^ = 0.701) and ABILITY × TIME interaction (*F*_(3,81)_ = 3.646, *p* = 0.016, partial η^2^ = 0.119). *Post hoc* paired-sample *t* test showed that the difference in rating between good and bad confederates was significant at each time point (all *p* < 0.01). Interestingly, although participants were able to detect the different ability levels, this has no effect on their Other Decision, with a regression showing no effect of agent ability nor changes in the use of shared evidence.

### Imaging results

#### Sampling phase

In this study, we were foremost interested in neural activity associated with two separate event types, occurring at different times in the trial. First, we investigated the sampling period, when participants accumulated evidence for estimating the state of the world, as well as the avatar's knowledge of the world. Second, we considered the Self and Other oriented decisions, dissociating the retrieval of individual knowledge about the state of the world from predictions about the avatar's knowledge.

**Figure 2.**
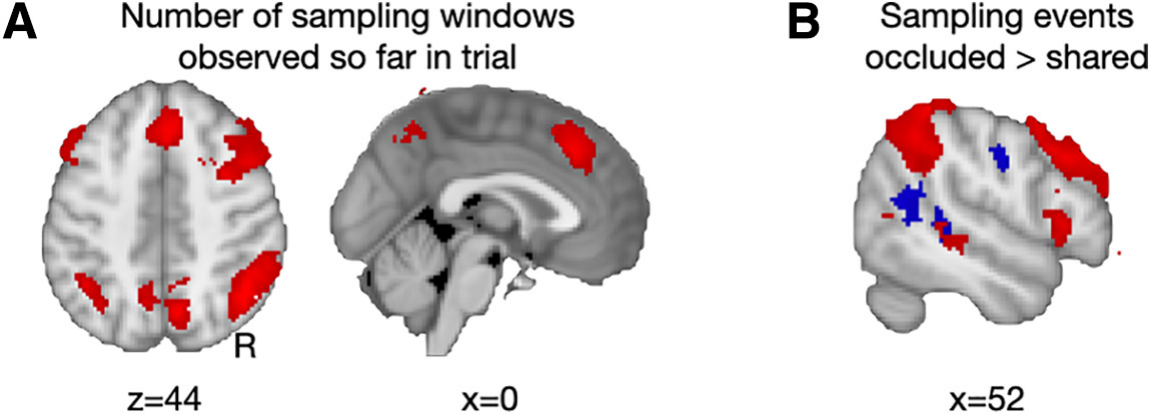
Imaging results—sampling phase. ***A***, Number of sampling windows observed so far in a trial was associated with an increase in activation in a widespread, predominantly parietal-frontal, network (red). ***B***, Sampling occluded events activated posterior STS and TPJ (blue), while increasing sampling number led to activity elsewhere (red).

When searching for increases in activation over the course of the sampling period, we found a large bilateral parietal-frontal network, encompassing the ventral banks of the intraparietal sulcus and middle frontal gyrus extending into the lateral frontal pole (FPl), but also the MFC (dorsal anterior cingulate cortex/8m) and medial parietal cortex (Fig. 2; Table 1). Smaller activations were seen in the right frontal operculum and right middle temporal gyrus. Thus, we found the expected results that parietal signals scale with sample number (i.e., the sequential integration process across both occluded and shared samples).

**Table:** Peak activations: sampling phase–sampling number

Anatomical region	MNI coordinates			Maximum *Z* value
	*x*	*y*	*z*	
Middle frontal gyrus	46	42	16	6.08
Inferior parietal lobule/intraparietal sulcus	44	−60	50	6.39
Visual cortex	22	−60	10	4.61
Inferior parietal lobule/intraparietal sulcus	−32	−54	36	5.08
Medial parietal cortex	8	−68	48	5.61
Anterior PFC	−30	56	−4	4.58
Visual cortex	−20	−82	4	4.35
Cerebellum (crus I)	−30	−58	−36	4.76
Middle temporal gyrus	58	−32	−4	4.43
Cerebellum (lobule VIIIa)	34	−44	−50	4.34

However, what we were most interested in during the sampling period was the difference between both types of sampling events. Thus, we looked at the difference between sampling of occluded compared to shared evidence. This showed increased activation in superior temporal sulcus extending into the temporoparietal junction area (TPJ) (Mars et al., 2012), medial and inferior parietal cortex, bilateral fusiform cortex, and dorsomedial frontal cortex in the pre-supplementary motor area (pre-SMA) when samples were occluded for the confederate (Fig. 2; Table 2). Additionally, we tested for the effect of the amount of evidence shown in the occluded and shared sample events, but found no further modulation of activity during the sampling period by evidence strength.

**Table 2.** Peak activations: sampling phase–occluded > shared events

Anatomical region	MNI coordinates			Maximum *Z* value
	*x*	*y*	*z*	
Fusiform gyrus	28	−46	−18	5.83
Superior temporal sulcus	64	−46	10	4.23
Medial parietal cortex	10	−62	44	4.69
Central sulcus	−40	−14	38	3.92
Inferior parietal cortex/intraparietal sulcus	−54	−42	56	4.26
Pre-SMA	−12	14	64	4.49
Precentral gyrus	62	0	20	1.35

#### Decision phase

Our main goal in this experiment was to investigate how regions of MFC are involved in translating the accumulated evidence into a concrete action, and whether this differs based on whether the action needs to be based on knowledge about the world or on information known to a task partner.

**Figure 3.**
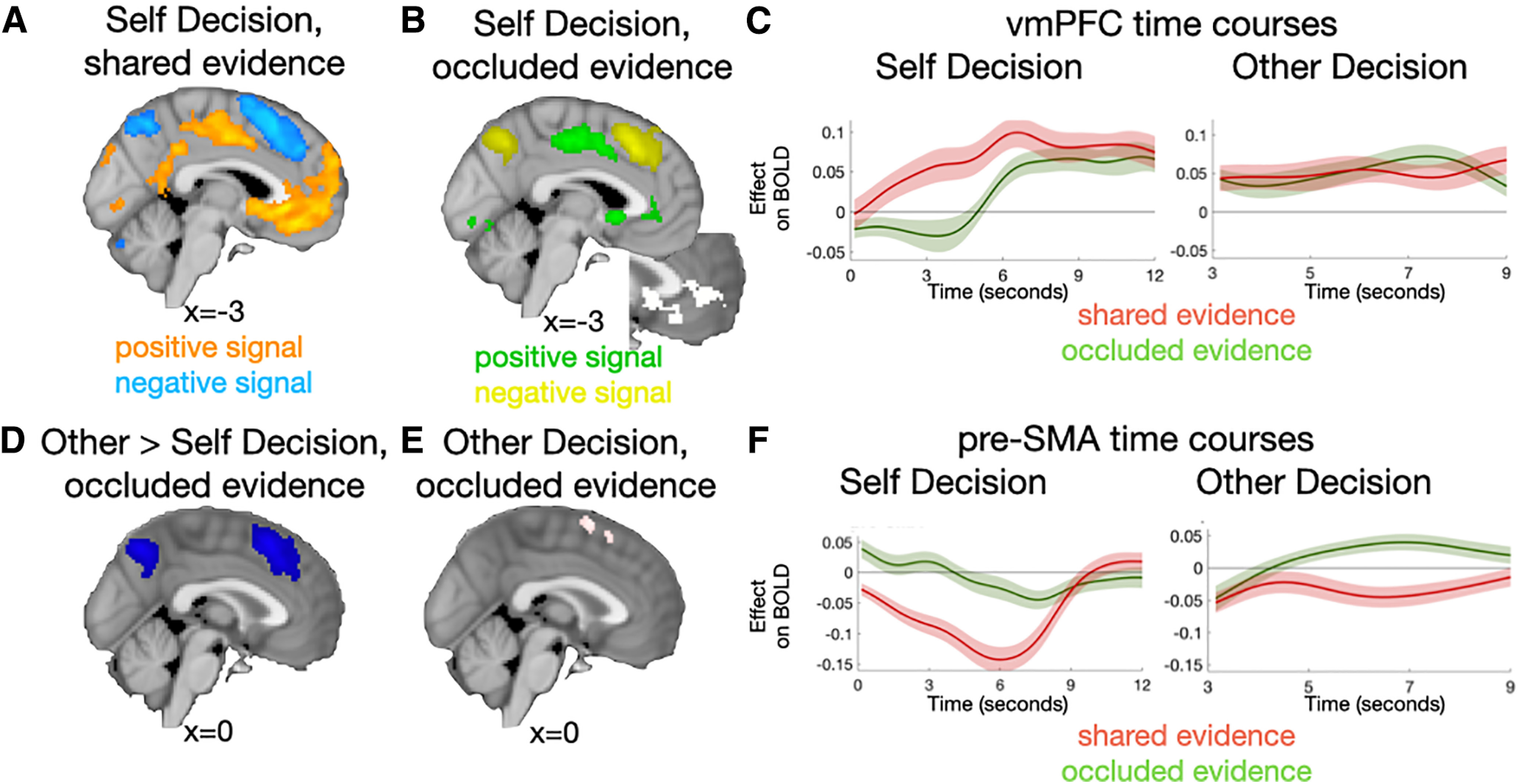
Imaging results—decision phase. Medial frontal activation during the Self Decision (***A***,***B***) and Other Decision (***D***,***E***). ***B***, Inset, Activation resulting from conjunction analysis between all evidence in the Self and Other Decisions (cluster threshold *Z* > 2.3). Time courses of vmPFC (***C***) and pre-SMA (***F***) during both Self and Other Decisions reflect a regression of the shared (red) and occluded (green) evidence on the BOLD time series. Time zero is the moment of presentation of the Self Decision and Other Decision choices on screen, respectively.

First, we tested for expected effects of evidence strength on deciding about the state of the world (i.e., are there more blue or red dots overall) during the Self Decision. We found strong effects of evidence strength in ventromedial PFC (vmPFC, including areas 14m, 11m, and 25) and posterior MFC (area 23ab and extending into caudal cingulate zone and posterior rostral cingulate zone). BOLD signal intensity decreased as evidence strength increased (“inverse decision value”) in dorsomedial PFC (dmPFC, extending from dorsal anterior cingulate into the pre-SMA) for both shared (Fig. 3*A*; Table 3) and occluded (Fig. 3*B*; Table 4) evidence. This was expected because, in Self Decisions, there is no difference in how the shared and occluded evidence should affect choices. vmPFC and dorsal cingulate cortex have previously been associated with positive and inverse decision value signals, respectively (Boorman et al., 2009; Kolling et al., 2012), which fits with their opposite pattern of activation here. While there is still some debate about the exact function of both brain regions in decision-making, both signals have been proposed to be hallmarks of decision-making processes. The medial wall activations were mirrored in lateral frontal networks, including in the lateral part of the frontal pole.

**Table 3.** Peak activations: Self Decision–shared evidence

Anatomical region	MNI coordinates			Maximum *Z* value
	*x*	*y*	*z*	
*Positive signals*				
vmPFC	4	40	−12	4.88
Inferior parietal lobule	62	−24	22	5.10
Amygdala (superficial group)	−14	−4	−18	4.69
Inferior parietal lobule	−58	−28	22	4.82
Posterior MFC	−2	−8	42	4.50
Amygdala (superficial group)	20	2	−16	4.01
Superior parietal lobule	22	−44	66	4.02
Superior parietal lobule	−20	−46	70	4.08
Visual cortex (V2, V1)	−8	−94	16	3.85
Visual cortex (V4, V3V, V2)	−18	−76	−10	3.86
*Negative signals*				
Lateral and medial PFC	−46	8	38	5.96
Superior and medial parietal lobule	10	−72	60	5.41
Anterior PFC	−40	60	6	4.36
Cerebellum (crus I)	−6	−80	−30	4.30
Inferior frontal cortex	38	26	−4	4.34
Cerebellum (lobule VI)	32	−48	−36	4.17

**Table 4.** Peak activations: Self Decision—occluded evidence

Anatomical region	MNI coordinates			Maximum *Z* value
	*x*	*y*	*z*	
*Positive signals*				
Posterior MFC	−8	−28	44	4.72
vmPFC	4	16	−4	4.43
Putamen	24	4	−10	4.73
Superior parietal lobule	24	−44	66	4.64
Inferior parietal lobule	−56	−26	20	4.43
Inferior parietal lobule	52	−28	28	4.35
Superior parietal lobule	−22-	48	64	4.41
Precentral gyrus	−24	−16	58	3.86
Visual cortex (V2, V1)	14	−94	28	3.72
vmPFC	−8	56	4	2.05
Opercular cortex	−46	2	2	4.26
Visual cortex (V5)	48	−72	−2	3.61
*Negative signals*				
dmPFC	0	26	42	6.26
Medial parietal cortex	4	−66	46	5.41
Inferior parietal lobule	36	−56	44	5.24
Inferior frontal cortex	30	26	2	4.80
Inferior frontal cortex	−30	24	0	3.70
Cerebellum (crus II)	−36	−58	−44	3.96
Anterior PFC	32	62	16	4.50
Cerebellum (lobule VI)	26	−58	−34	3.80

These contrasts demonstrate that our paradigm allows us to differentiate the main effects commonly reported for the various MFC subdivisions during decision-making. However, we were most interested in the activation patterns when participants had to predict what others will do, instead of deciding about the state of the world overall. When deciding about others' view of the world during the Other Decision, the decision had to be based on the shared evidence only, while ignoring the occluded samples.

Therefore, we next investigated whether the reported regions of MFC dissociate between the Self Decision and the Other Decision, where privileged information presented only to the participant in the occluded evidence should be ignored. We compared the effect of occluded evidence in Self Decisions, when it should be just a straightforward part of the evidence integration process, with occluded evidence in the Other Decisions, where it should be ignored. Doing so revealed a large effect in dmPFC (including dorsal anterior cingulate cortex and pre-SMA) (Fig. 3*D*; Table 5), but no significant differences in vmPFC. Outside the MFC, this change in occluded evidence signals based on decision context was also evident in a widespread parietal-frontal network.

**Table 5.** Peak activations: decision phase, occluded evidence—Other > Self Decision

Anatomical region	MNI coordinates			Maximum *Z* value
	*x*	*y*	*z*	
dmPFC	−2	33	50	6.05
Inferior parietal cortex/intraparietal sulcus	38	−76	42	4.47
Medial parietal cortex	−2	−62	46	5.69
Inferior parietal cortex/intraparietal sulcus	−34	−76	42	4.14
Inferior frontal cortex	−30	24	0	4.88

From this whole-brain comparison contrast, it is possible to infer that dmPFC is successfully ignoring the occluded information when making decisions, while vmPFC is still including it in its consideration as it is part of what the person knows to be the true state of the world. However, alternatively, dmPFC could be actively suppressing occluded evidence to prevent such knowledge to contaminate one's judgment about the other person. Given that both options are possible when looking at a comparison contrast (a lack of signal or a reversal of signal), we wanted to explicitly test whether occluded evidence might activate dmPFC, despite it being deactivated with more choice evidence when judging the whole state of the world. Thus, we tested for activation with occluded evidence in Other Decisions on the whole-brain level. Here we found activation of the pre-SMA, partially overlapping with the dmPFC cluster reported in the previous analysis (Fig. 3*E*; Table 6). dmPFC thus not only decreased in activity with increased shared and occluded evidence during Self Decisions, but importantly increased its activity right after with increasing occluded evidence in the Other Decision, which is the exact opposite effect.

**Table 6.** Peak activations: Other Decision—occluded evidence

Anatomical region	MNI coordinates			Maximum *Z* value
	*x*	*y*	*z*	
dmPFC	−4	8	62	3.60
Middle frontal gyrus	24	28	36	3.63

This dissociation between ventral and dorsal part of the MFC is also evident in the time courses of activation during the decision phase. Both shared and occluded evidence affects activation in vmPFC during the Self Decision (convolved HRF effect shared *t*_(27)_ = 5.2127, *p* < 0.001; occluded *t*_(27)_ = 4.3887, *p* < 0.001), and this activation is sustained during the Other Decision (shared *t*_(27)_ = 3.5557, *p* = 0.0014; occluded *t*_(27)_ = 4.3634, *p* < 0.001) (Fig. 3*C*). This consistent activation of vmPFC is also emphasized by a conjunction analysis (Nichols et al., 2005) of all evidence during the Self and Other Decisions (Fig. 3*B*, cluster threshold *Z* > 2.3). In contrast, the time courses of pre-SMA differ dramatically between the Self and Other Decisions, with a large negative effect of occluded evidence during the Self Decision (*t*_(27)_ = −2.1754, *p* = 0.0385) shifting to a positive effect during the Other Decisions (*t*_(27)_ = 2.2120, *p* = 0.0356; Fig. 3*F*), while the sharp negative signal for the shared evidence during the Self Decision (*t*_(27)_ = −6.6359, *p* < 0.001) is somewhat reduced during the Other Decision (*t*_(27)_ = −2.2289, *p* = 0.0343. We note that, since the vmPFC ROI time course was selected based on the shared evidence in the Self Decision contrast and the pre-SMA ROI time course on the Other Decision occluded evidence, we report their convolved statistics for completeness sake and use the time courses as illustration as neither is an unbiased test of significance.

#### Posterior-frontal interactions

During the Other Decision, the participant had to consider that some of the evidence they had was privileged as it was occluded for the other person. Above, we demonstrated that areas around the TPJ were more active when a sample was occluded for another person during the sampling phase. This suggests that TPJ might be engaged with considering information that is socially relevant so that the participant can make a prediction about the other person's behavior later. If this is true, it would be reasonable to expect that TPJ interacts with the relevant prefrontal regions during this sampling and during other decisions. We tested this notion using a psychophysiological interaction (O'Reilly et al., 2012) analyses that test for increased functional connectivity between areas linked to a psychological variable.

**Figure 4.**
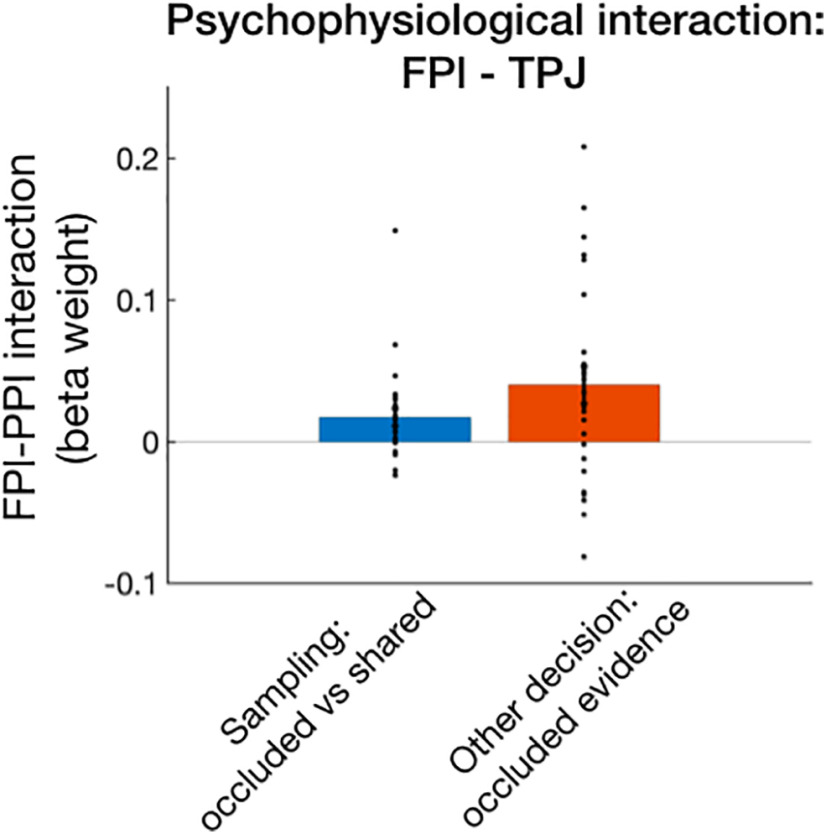
Psychophysiological interaction (PPI) reflecting interaction between FPl and TPJ. The two areas' time courses were significantly more correlated in the sampling phase when presented with occluded compared with shared evidence, and during the decision phase reflected occluded evidence in the Other Decision.

During the sampling phase, TPJ indeed had increased connectivity with the FPl when a sample was occluded compared with shared (Fig. 4; *t*_(27)_ = 2.7884, *p* = 0.0096). Importantly, FPl is frequently implicated in value-based decision-making and learning (e.g., Rushworth et al., 2011). During the other decision, TPJ-FPl connectivity was also increased as a function of occluded evidence during Other Decisions (*t*_(27)_ = 3.0849, *p* = 0.0047), but not during Self Decisions (*t*_(27)_ = 1.6979, *p* = 0.1010).

## Discussion

Investigation of the computational and neural mechanisms underlying social cognition is challenging because of the open-ended nature of human interactions and the recursive thought processes that might underlie them. Here, we designed a task to isolate one of the most fundamental aspects of social interaction (i.e., knowing how to use privileged and shared information). By objectively quantifying knowledge used during social and personal decision-making, we differentiated the roles of two prefrontal brain regions in making decisions about oneself and predicting other people's behavior based on shared information, but not information about the world privileged to the participant.

There are at least two ways a person might solve our social sequential evidence accumulation and decision task. They could simply accumulate two evidences in parallel: one for the shared and occluded evidence combined to make “Self Decisions” and one only for the shared evidence to make predictions about the other person. Alternatively, a person could accumulate all evidence together but keep track of or otherwise tag occluded evidence to be able to construct predictions about other agents when necessary, by subtracting such privileged information from one's own expectation of the world. The advantage of the second approach is the ability to rely on most of one's own knowledge to construct only one world model and simply modify it to predict the behavior of others. This ego-centric perspective, which is modified to take other people's perspectives, has the added advantage that it allows simulation of many different types of others by simply modulating other aspects of one's world view. This would be consistent with the observation that egocentric biases grow as people are more cognitively loaded (Vogels et al., 2015; Cane et al., 2017), suggesting that egocentric coding of information is the least effortful. It does have the disadvantage of being vulnerable to ego-centric biases in the form of incomplete perspective taking for people that are different from one's self. We have some evidence of this in participants' inability to completely suppress occluded information when predicting other people's behavior. We argue that the neural data also support such a view.

During the sampling phase, when the participant is simply monitoring and integrating evidence for their own judgment about the world, we found increasing activation in a widespread parietal-frontal network. Such patterns of activation are expected based on these regions' general role in action selection and have been shown during similar task settings (Liu and Pleskac, 2011; Pisauro et al., 2017). Importantly, already at the sampling stage, activation of posterior temporal cortex around the posterior TPJ increased for occluded compared with shared samples. It has been argued that parts of the superior temporal cortex and the posterior part of TPJ are predominantly activated during social information processing in both humans and nonhuman primates (Saxe, 2006; Mars et al., 2013; Schurz et al., 2014). However, although it is possible to demonstrate distinct activation patterns for the various subregions of posterior temporal cortex (Schurz et al., 2017), capturing their precise contribution has remained difficult. Our results directly speak to this question as TPJ was activated by the presentation of information that another person does not have access to. This suggests that TPJ's role in social cognition might be to signal the elements of another person's world that differ from one's own or to represent the discrepancy between the true and other's state of the world. In this context, it is interesting that we observed a psychophysiological interaction between TPJ and the FPl, which has been associated with processing counterfactual information (Boorman et al., 2009; Koechlin, 2014).

During the Self Decision, we found activations that would be expected in this task without any social manipulation. Both evidence positive and negative brain regions in parietal and lateral and medial PFC activated the way they do in value-based decision-making (Boorman et al., 2009; Scholl et al., 2015). However, during the Other Decision, activation in the decision network changed in revealing ways in different regions. vmPFC reflected the person's egocentric predictions about the world consistently, regardless of whether participants had to make a judgment on their state of the world or the other persons. dmPFC, on the other hand, always signaled the evidence relevant to the decision at hand only, suggesting it could flexibly adjust to only take the currently decision-relevant information into account, much like participants did behaviorally. Pre-SMA changed from being less active with increasing occluded evidence in the Self Decision (this evidence supported faster decision-making) to being more active with increasing occluded evidence (here this evidence needs to be ignored or even suppressed to make predictions about the other person). In other words, pre-SMA might have played a role in ensuring accurate predictions about others by helping constructing such beliefs through inhibition of irrelevant information. This pattern of activation is certainly consistent with the proposed role of this area in changing behavior and suppression of prepotent responses based on external cues (Nachev et al., 2007; Mars et al., 2009). Using connectivity analyses, we furthermore identified frontal polar connectivity with TPJ as potentially relevant in monitoring and retrieving the occluded evidence that needs to be excluded from other predictions.

It has been argued that the well-established mechanisms of reinforcement learning can be applied to social information processing by changing the frame of reference in which the information is processed (Behrens et al., 2009). This approach has led to the suggestion that medial wall regions do not necessarily dissociate between “social” and personal or “nonsocial” decision problems, instead processing information to guide current actions or to update a latent model of the current world (Nicolle et al., 2012). Indeed, there is a lively debate about whether there are uniquely social decision-making areas (Rushworth et al., 2013; Lockwood et al., 2020). Many of the activation patterns of prefrontal regions we observed here are consistent with roles attributed to them in agent-based decision-making. In general, such decision-making paradigms allow better control of the experimental variables, enabling careful quantification of brain signals related to choice using computational cognitive models. They also allow for the dissociation of different decision variables and the characterization of unchosen options and irrelevant information. As a case in point, Scholl et al. (2015) were able to demonstrate that a network of dmPFC and frontal pole together is involved in suppressing maladaptive interference of irrelevant but real reward experiences when making value-guided decisions. Importantly, our paradigm shows that a very similar network is activated when one has to suppress egocentric knowledge to infer the state of mind of another person. Scholl et al. (2015) also found vmPFC as the counterweight, linking it to decisions that were driven by egocentric or experienced reward events, whereas dmPFC and frontal pole were linked to better usage of the abstract knowledge that should optimally guide behavior. Thus, our data point to the conclusion that the same networks involved in personal decision-making are involved in making predictions about other people. This is consistent with the view that human social cognition has its basis in the ability to make and evaluate decisions that is present in other species (Frith and Frith, 2012).

Our data could help bridge the gap between the personal, egocentric decision-making literature concerned with how people optimize their own rewards and the social literature that is more interested in how we understand other people and predict their behavior. Previously, in social decision-making, the distinct roles of vmPFC and dmPFC have often been interpreted in terms of a dissociation between processing emotional value and attribution of intentional state (Amodio and Frith, 2006; Saxe, 2006). This interpretation would give those brain regions very different functions in social compared with personal contexts. Our present study offers a perspective that could reconcile social and personal observations.

Overall, our data offer some evidence for the notion that, instead of there being a dedicated social network that in one step simulates and predicts other people's behavior from scratch only using social information, social predictions might be a two-step process. The first step is the retrieval of the relevant egocentric knowledge about the world as you would for personal choices, which some brain regions, such as the vmPFC, hold naturally when making decisions about the self. The second step involves contextualization and modification of this knowledge by one's knowledge of the world view of the other person. Neurally, regions, such as TPJ, might supply information about what the other does not know. dmPFC contextualizes and modifies the prediction by this context, as it would for personal decisions that require contextualization or suppression of irrelevant information. Psychologically, our data suggest that predictions about other people can start from one's own egocentric perspective, which is then altered, at least if one predicts their behavior based on a substantially shared world. This perspective is further supported by participants' inability to completely ignore their privileged knowledge when predicting others.

The current paradigm of using evidence accumulation in a social setting allowed us to successfully dissociate signals related to processing knowledge about the world and about others. The paradigm has the potential to be further developed to investigate other signals of relevance to social and communicative information processing, including dealing with a lack of knowledge on the part of the participant and overcoming biases in the information processing of the confederate or explicitly learning about the different confederates' abilities. Moreover, it would be interesting to investigate a setting in which the two participants are working toward a common goal, as would be the case in cooperative social interactions. Finally, although we ran analyses coding evidence in terms of whether it reaffirms or changes the current decision, we found no strong signals relating to these effects. This might be because participants are not making decisions at that stage yet, but instead only trying to track the overall evidence levels to choose later. The precise moment of the decision might also be probed in the future using more time-sensitive measures, including neural perturbation techniques.
